# Gait Parameters Can Be Derived Reliably and Validly from Augmented Reality Glasses in People with Parkinson’s Disease Performing 10-m Walk Tests at Comfortable and Fast Speeds

**DOI:** 10.3390/s25041230

**Published:** 2025-02-18

**Authors:** Pieter F. van Doorn, Daphne J. Geerse, Jara S. van Bergem, Eva M. Hoogendoorn, Edward Nyman, Melvyn Roerdink

**Affiliations:** 1 Department of Human Movement Sciences, Faculty of Behavioural Sciences, Vrije Universiteit Amsterdam, Amsterdam Movement Sciences, 1081 BT Amsterdam, The Netherlands; 2 Strolll Ltd., Stafford ST16 2LP, UK; 3 Department of Nutrition and Movement Sciences, NUTRIM Institute of Nutrition and Translational Research in Metabolism, Faculty of Health, Medicine and Life Sciences, Maastricht University, 6211 LK Maastricht, The Netherlands; 4 Department of Nutrition and Movement Sciences, MHeNs Institute of Mental Health and Neurosciences, Faculty of Health, Medicine and Life Sciences, Maastricht University, 6211 LK Maastricht, The Netherlands

**Keywords:** augmented reality, Parkinson’s disease, 10-m walk test, gait speed, concurrent validity, test-retest validity, face-validity

## Abstract

The 10-m walk test (10MWT) is a stopwatch-based clinical mobility assessment. To better understand mobility limitations, 10MWT test completion times may be complemented with gait parameters like step length. State-of-the-art augmented reality (AR) glasses can potentially do this given their unique 3D-positional data from which gait parameters may be derived. We examined the test-retest reliability, concurrent validity, and face validity of gait parameters derived from AR glasses during a 10MWT in 20 people with Parkinson’s disease, performed at self-selected comfortable and fast-but-safe walking speeds. AR-derived 10MWT completion times and gait parameters (mean step length, cadence, and maximal gait speed) were compared across repetitions and with lab-based (Interactive Walkway) and clinical (stopwatch) reference systems. Good-to-excellent test-retest reliability statistics were observed for test completion times and gait parameters for all systems and conditions alike. Concurrent validity was demonstrated between AR, lab-based, and clinical references for test completion times (good-to-excellent agreement: ICC > 0.879) and gait parameters (excellent agreement: ICC > 0.942). Face validity was confirmed by significant differences in test completion times and gait parameters between speed conditions in a-priori expected directions. These findings support the conclusion that gait parameters can be derived reliably and validly from AR glasses in people with Parkinson’s disease.

## 1. Introduction

Parkinson’s disease is a progressive, neurodegenerative disorder with an estimated rising global prevalence of 1.51 cases per 1000 individuals [1]. This condition has a profound impact on mobility and is characterized by gait impairments, such as reduced step length and gait speed [2]. In 2019 alone, Parkinson’s disease accounted for 5.8 million disability adjusted life years (DALYs) worldwide, underscoring its significant global burden [3]. Gait impairments are particularly critical, serving as key markers of disease progression and functional decline. Among the various clinical assessments available, the 10-m walk test (10MWT) stands out as a widely used and validated tool for evaluating gait speed, an essential indicator of mobility, fall risk, and overall health status [4]. Consequently, the 10MWT has become a cornerstone of Parkinson’s disease management, aiding in the monitoring of disease progression, tailoring of treatment strategies, and evaluation of therapeutic interventions [5].

Traditionally, the 10MWT is performed in the clinic with test completion time, as registered by the clinician with a stopwatch, as the main outcome. However, this method may limit the clinician from observing the patient for safety and visual assessment. Moreover, test completion time only provides a single value, potentially masking clinically relevant variations in gait parameters like step length and cadence or other aspects affecting test completion time, such as difficulty with gait initiation or freezing of gait. Therefore, digital health technology in the form of various sensor-based 10MWTs has been developed to automate the test and derive clinically meaningful gait parameters such as step length, maximum gait speed, and cadence from the 10MWT [6].

Augmented reality (AR) glasses, such as Microsoft HoloLens 2 and Magic Leap 2, are promising digital health technology devices as they can be used as a motion-registration system, utilizing their unique visual Simultaneous Localization and Mapping (vSLAM) algorithms to determine real-time 3D position and orientation time series of the glasses in space [7]. Additionally, they may serve as a potential AR assessment tool to (self-)administer clinical tests, using holographic content to set test constraints (e.g., present a holographic line at the start and end of the 10 m and using test and start instructions to guide and standardize trial execution). Geerse et al. (2020) previously showed that Microsoft HoloLens 1 provided reliable and valid data for quantifying various spatiotemporal gait parameters for the 10MWT executed at a self-selected comfortable speed in people with Parkinson’s disease and for self-selected slow, comfortable, and fast speeds in healthy adults [8]. However, parameters were only derived between 1 m and 7 m of the 10-m walkway and lacked a direct comparison with the clinical 10MWT standard: test completion time. Furthermore, no face validity evaluation of known-speed effects on gait parameters was performed in people with Parkinson’s disease.

The aim of this study was to comprehensively evaluate the reliability and validity of 10MWT gait parameters derived from state-of-the-art AR glasses (Magic Leap 2, Microsoft HoloLens 2) in people with Parkinson’s disease, performed at self-selected comfortable and fast-but-safe speeds, with two repetitions each. We concurrently registered AR 3D positional data from AR glasses and 3D full-body kinematics from a markerless motion-registration system (Interactive Walkway, lab-based reference system) from which gait parameters were calculated. Additionally, test completion times from a stopwatch (clinical reference system) were registered. The specific objectives were to determine the following:

1. Test-retest reliability for all systems and conditions separately (What are the limits of agreement of derived 10MWT parameters over repetitions?);
2. Concurrent validity of AR 10MWT parameters against reference systems (How well do 10MWT parameters derived concurrently from AR and reference-system data agree?);
3. Face validity of AR 10MWT parameters across comfortable and fast-but-safe execution of the 10MWT (Do the derived AR gait parameters vary in a-priori known directions?).

We expect that 10MWT parameters derived from state-of-the-art AR glasses data are reliable for test-retest, agree well with those derived from reference systems, and vary in a-priori known directions over the walking speed conditions (i.e., longer step length, higher cadence, higher maximal speed from comfortable to fast-but-safe speed conditions [9,10]. Test-retest reliability and concurrent validity considered together, we specifically expect narrower limits of agreement for between-systems differences than for within-system(s) repetitions (i.e., better between-systems agreement than between-repetitions agreement within systems) [8]. Likewise, for concurrent validity and face validity together, we expect well-detectable within-system differences in 10MWT parameters in a-priori known directions between self-selected comfortable and fast-but-safe speeds and that these differences are greater in magnitude than between-systems biases [8].

## 2. Materials and Methods

### 2.1. Subjects

A convenience sample of 20 people with Parkinson’s disease participated in this study. Inclusion criteria were an age of 18 years and above, capable of walking independently for over 30 min, and a cognitive function sufficient to understand test instructions. Exclusion criteria were any other neurological or orthopedic conditions significantly interfering with gait function, visual or hearing impairments (after corrective aids), and severe visual hallucinations or illusions. Ethical approval was obtained from the Medical Research Ethics Committees United, the Netherlands (R22.076, NL82441.100.22, 21 November 2022), and the research was carried out in accordance with the principles of the Declaration of Helsinki. Written consent was obtained from all participants prior to this study. All participants maintained their daily medication routine. Participant characteristics are summarized in Table 1.

### 2.2. Experimental Set-Up and Procedures

All participants participated in one visit to the gait laboratory at the Vrije Universiteit Amsterdam. Participants performed a static 10MWT in two conditions: (1) at their own self-selected Comfortable Walking Speed (CWS) and (2) as fast as safely possible without running (FWS). Briefly, the participant stood still at the 0-m line and started walking towards the 10-m line after countdown by the instructor [14]. The test was completed when the participant crossed the 10-m line with either of their two feet. To evaluate test-retest reliability and face validity for AR data-derived gait parameters, all participants performed the 10MWT in the following fixed order in a single measurement session: CWS trial 1, FWS trial 1, CWS trial 2, and FWS trial 2, with sufficient rests in between. 

### 2.3. Data Acquisition

**Figure 1:**
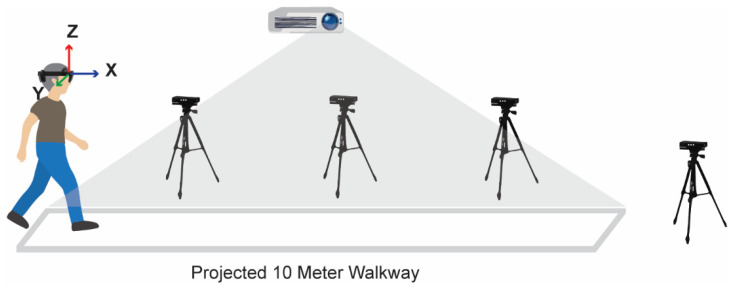
Schematic of the experimental setup. The participant is performing the 10MWT while wearing AR glasses. The Interactive Walkway, comprising a projector and four Kinect cameras, is used as a lab-based reference motion-registration system.

Measurements during the 10MWT were performed with HoloLens 2 (N = 11) or Magic Leap 2 (N = 9) AR glasses, block-randomized over participants, with concurrent registrations with the Interactive Walkway (IWW; a lab-based reference for full-body 3D markerless motion tracking [15]) and a stopwatch (a clinical reference to manually register test completion time). HoloLens 2 and Magic Leap 2 are state-of-the-art AR glasses that are both capable of tracking their 3D position and orientation relative to their surroundings at sampling rates of approximately 30 Hz and 60 Hz, respectively. The 3D position time series provides valuable features for identifying gait parameters like step length, stride length, gait speed, and cadence [8]. The Interactive Walkway is a 10-m walkway with a projector designed for walking-adaptability assessments like sudden obstacle avoidance [16]. It utilizes four integrated Microsoft Kinect v2 sensors to track in a markerless manner the 3D positions of 25 body points [17,18,19], including the head, spine, shoulder, and feet, along an 11–12 m path with data sampled at approximately 30 Hz. The validated Interactive Walkway setup used in this study aligns with the one described by Geerse et al. [8]. The Interactive Walkway has demonstrated good validity for walking-adaptability and gait parameters compared to sub-millimeter precision marker-based systems [20]. The Interactive Walkway has been used to assess walking adaptability and gait characteristics in healthy adults, as well as in individuals with stroke and Parkinson’s disease [16], showing strong validity in distinguishing between groups based on gait parameters like gait speed, step length, and cadence. In this study, the ultra-short-throw projector (EPSON EB-585W) of the Interactive Walkway was only used for presenting the 10-m walkway path (Figure 1).

### 2.4. Data (Pre-)Processing

**Figure 2:**
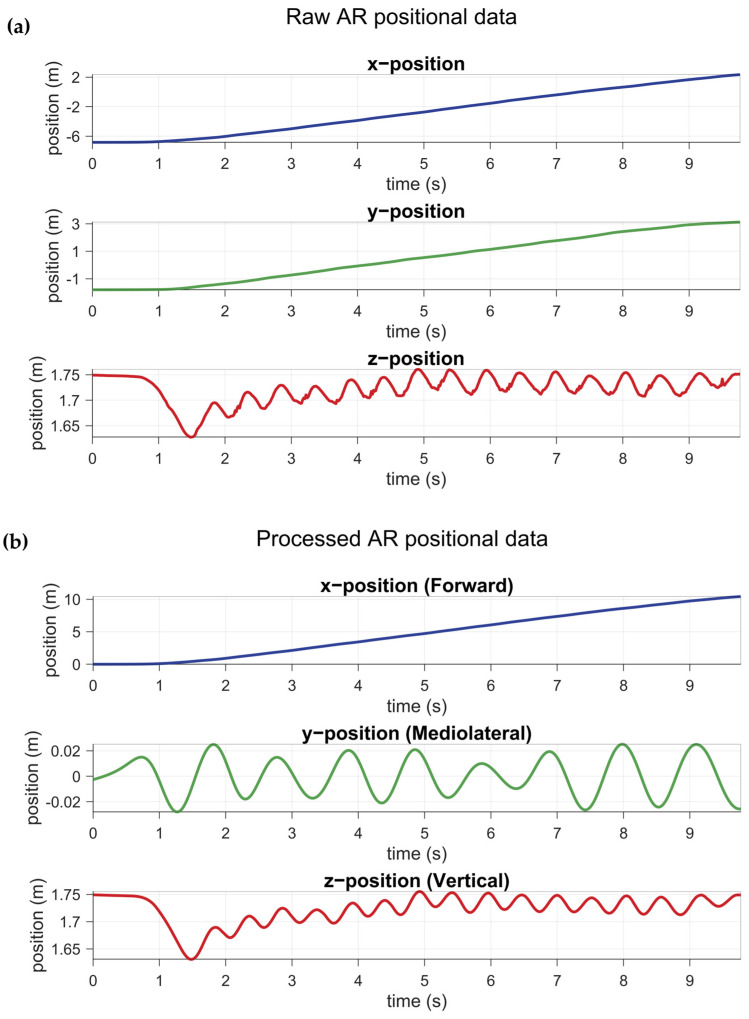
A typical example of raw (**a**) and processed (**b**) 10MWT AR positional data showing the effects of reorienting, recentering, and filtering.

The spatial coordinate system from the AR glasses was realigned to a left-handed coordinate system, where a positive X-axis points forward, the positive Y-axis points to the right, and the positive Z-axis points upward (Figure 1). To this end, the forward direction was defined by first identifying an initial walking segment based on the speed calculated from the X and Y position using the Pythagorean theorem. This walking segment was then used to realign the trajectory along the forward direction using linear regression: X and Y position data were reoriented to a new coordinate system, with the X-axis pointing forward and the Y-axis perpendicular to the X-axis. Lastly, initial offsets were removed from X and Y positional data by subtracting the first value from the data. This reorientation and centering are visualized in Figure 2.

#### 2.4.1. Test Completion Time

**Figure 3:**
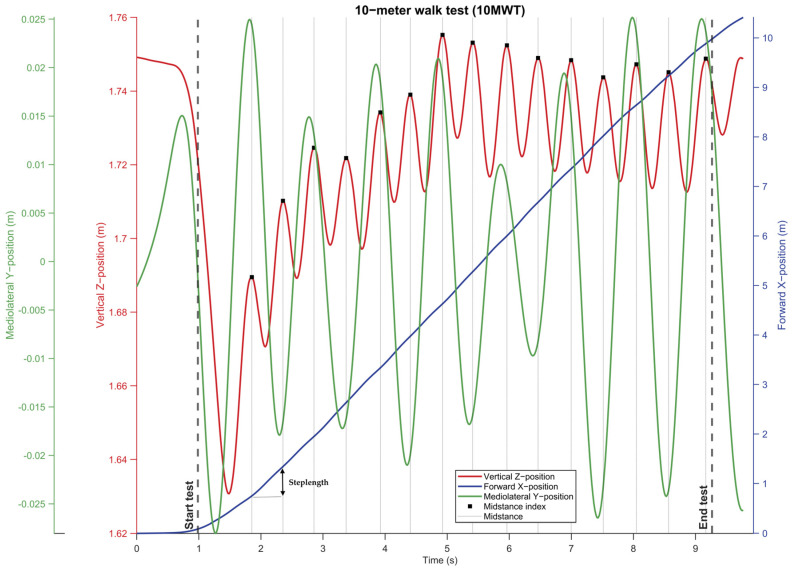
A typical example of 10MWT AR positional data. The vertical Z position (red line) was used to detect the peaks, representing indices at or around midstance (dotted vertical lines). From the mediolateral Y position data (green line), right (peak at or around midstance), or left (valley at or around midstance) steps were classified. Features of the forward X position (blue line) were used to determine the step length (from midstance-to-midstance index, visualized with the double-headed arrow for a single step) and the start and end of the 10MWT test (dashed lines).

To identify the test completion time of the 10MWT from AR data, the start index was pragmatically determined as the first forward displacement of 10 cm while the end index was determined as the displacement of 9.88 m (accounting for the 10 cm displacement at the start and an uncertainty margin of 2 cm; Figure 3). Finally, test completion time was calculated by the difference in time elapsed from the start index to the end index (in seconds). 

#### 2.4.2. Step-Detection Algorithm

Characteristic peaks in the vertical Z position data of head-worn AR glasses are representative of indices of midstance/midswing in the gait cycle [21]. To detect these peaks, the vertical Z position data were smoothed using a Savitzky-Golay filter with a polynomial order of 2. The filter’s window length was tailored to each participant by estimating the main frequency of their vertical Z position data during the walking segment, derived from a Fast Fourier Transform (which approximately corresponds to the step frequency). This approach reduced high-frequency noise while avoiding over-smoothing (Figure 2). Local maxima in the smoothed vertical Z position data were detected as indices at or around midstance using the find peaks function in Matlab. We constrained the temporal distance between detectable peaks by 60% of the step frequency and used a peak prominence of 0.001 (Figure 3).

#### 2.4.3. Gait Parameters

Gait parameters were derived from features in the 3D positional AR data. Specifically, the step length for each step was determined as the displacement in the reoriented forward X-direction between consecutive step locations, defined as X positions at midstance indices (Figure 3); mean step length was then taken for further statistical processing. The cadence was calculated by determining the number of steps (i.e., the number of detected midstance indices minus one) divided by the time elapsed between the first and last midstance index in minutes. The maximal gait speed was calculated as the maximum of the derivative of the forward X position over time. To reduce high-frequency noise in the calculated speed signal, a moving average filter was applied with a window size of one second from start to finish.

Left and right steps were classified using the mediolateral Y position at the identified midstance indices. First, the Y position data were linearly detrended to remove any long-term trends. Second, a bandpass filter was applied, with cutoff frequencies set to unveil features associated with mediolateral head sway during walking [21]. The lower cut-off frequency was one-quarter of the approximated step frequency (see Section 2.4.2), and the upper cut-off frequency corresponded to the approximated step frequency (Figure 2). Third, the filtered Y position data were temporally aligned with the vertical Z position data by calculating the phase difference using a Hilbert transform [22]. This alignment was achieved by extracting the instantaneous phases of both signals, computing the phase difference, and applying a temporal shift to the mediolateral position data to align its phase with the vertical position data. Following alignment, peaks and valleys in the filtered mediolateral position time series, corresponding to right and left steps, were identified, respectively. Each midstance index was then classified as a left or right step by identifying the nearest valley or peak in the aligned mediolateral signal, respectively (Figure 3). From the 559 right steps, 545 were identified correctly as right steps with AR data, whereas from the 548 left steps, 540 were identified correctly as left steps with AR data. The true right rate was 97.5%, the true left rate was 98.5%, and the balanced accuracy for left/right step detection from AR data during the 10MWT was 98.0%. Interestingly, 20 of the 22 incorrectly identified steps belonged to a single participant, showing abnormal head movements associated with dyskinesia. To reduce the number of misclassified steps, an enforced step alternation constraint was added to the classification, starting from the first classified step.

### 2.5. Statistical Analysis

Test-retest reliability (i.e., the absolute within-system agreement between trials, separately for all conditions) and concurrent validity (i.e., the absolute agreement between systems, separately for all conditions, using the first trial per speed condition) were evaluated with the intraclass correlation coefficient for absolute agreement ICC_(A,1)_. ICC values above 0.50, 0.75, and 0.90 represent moderate, good, and excellent agreement, respectively [23]. ICC values were substantiated with the bias (indicating significant systematic differences over repetitions and/or between systems) and the limits of agreement obtained with a Bland–Altman analysis [24]. Biases were evaluated using paired-sample *t*-tests under the verified assumption of normality for all trials using Shapiro–Wilk tests [25]. The face validity of AR-derived gait parameters was tested by evaluating if differences were found in a-priori known directions over speed conditions and compared to similar differences with reference systems. To this end, Condition (2 levels: CWS, FWS) × System (2 or 3 levels: AR, Interactive Walkway, Stopwatch) repeated-measures ANOVAs [26] were run for test completion time, mean step length, cadence, and maximal gait speed, again using the first trial per speed condition. Statistical analyses were run in Matlab version 2023b, with significance set at 0.05 and effect sizes reported as partial *η*^2^. All raw and processed data, including dependent variables used in the statistical analysis, can be found in the Supplementary Material.

## 3. Results

### 3.1. Test-Retest Reliability

The absolute agreement statistics (ICC, bias, limits of agreement) for both CWS and FWS conditions for all parameters are presented in Table 2. All ICC values were good and excellent for all systems alike. Statistically significant biases were observed, mainly for the CWS condition, with faster retests than test completion times for all systems. Also, some statistically significant biases were observed in the gait parameters, as detailed in Table 2. 

### 3.2. Concurrent Validity

The absolute agreement between AR-derived 10MWT completion times and the stopwatch completion times was good for CWS and excellent for FWS conditions (Table 3), with small-but-significant biases (~5% and ~3% of the mean, respectively). The absolute agreement between AR-derived 10MWT gait parameters and IWW-derived counterparts were excellent (ICC_(A,1)_ > 0.942; Table 3) for both CWS and FWS conditions, with small biases (<1.2% of the mean; the bias was only significant for CWS and FWS cadence; Table 3) and narrow limits of agreement; note that these between-systems limits of agreement were as expected all narrower than their within-system(s) counterparts over trial repetitions (compare Table 2 and Table 3). Finally, from a total of 1107 detected steps, all steps were classified correctly as left or right steps, yielding a 100% balanced accuracy for left/right step detection from AR data during the 10MWT.

### 3.3. Face Validity

**Figure 4:**
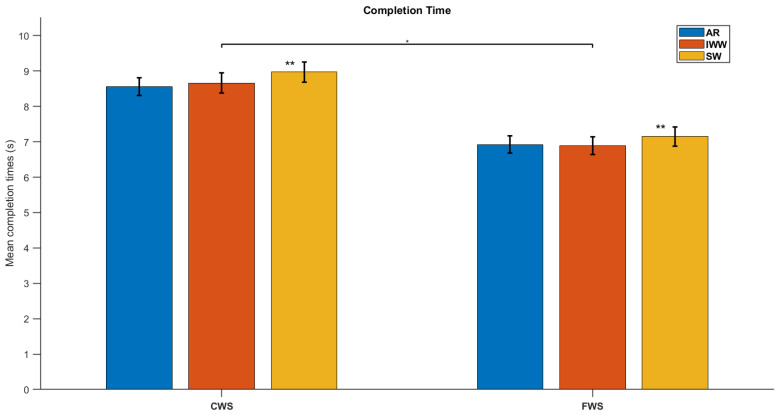
Mean values and their standard error of the mean of completion times from all systems (Augmented Reality glasses (AR), Interactive Walkway (IWW), and Stopwatch (SW)) for both conditions (self-selected Comfortable Walking Speed (CWS) and Fast Walking Speed (FWS)). * significant between condition effect, ** significant between system effects for SW with both AR and IWW.

**Figure 5:**
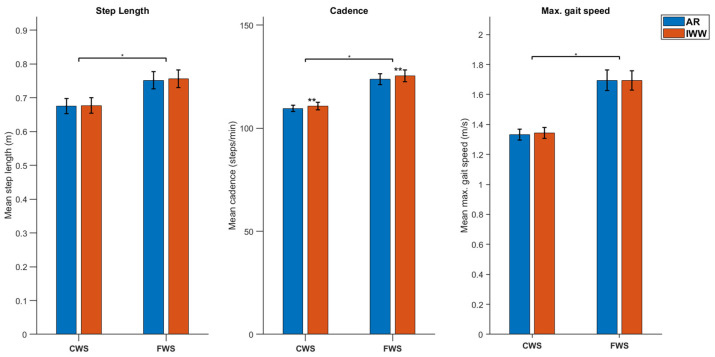
Mean values and their standard error of the mean of gait parameters derived from Augmented Reality glasses (AR) and the Interactive Walkway (IWW) for both self-selected Comfortable (CWS) and Fast-But-Safe (FWS) Walking Speed conditions. * significant between condition effect, ** significant between system effect.

For all systems, significant main effects of Condition were found in expected directions. Test completion time decreased significantly from CWS to FWS conditions (F(1.0, 19.0) = 98.1, *p* < 0.001, partial *η*^2^ = 0.838; Figure 4), while mean step length, cadence and maximal gait speed all increased significantly from CWS to FWS conditions (F(1.0, 19.0) = 38.2, *p* < 0.001, partial *η*^2^ = 0.668, F(1.0, 19.0) = 38.9, *p* < 0.001, partial *η*^2^ = 0.672, and F(1.0, 19.0) = 55.5, *p* < 0.001, partial *η*^2^ = 0.745, respectively; Figure 5).

For test completion time, also a significant main effect of System was found (F(2.0, 38.0) = 13.7, *p* < 0.001, partial *η*^2^ = 0.420), with significant post-hoc differences between stopwatch and AR systems and between stopwatch and Interactive Walkway systems, where in both cases test completion times were slightly longer with the stopwatch (Figure 4), a finding in line with the significant bias observed between these systems for both CWS and FWS conditions (Table 3). Likewise, in line with the significant between-systems biases for cadence (Table 3), also here a significant main effect of System was found (F(1.0, 19.0) = 14.3, *p* = 0.001, partial *η*^2^ = 0.430), albeit much smaller in magnitude and effect size than the effect of Condition on cadence (Figure 5). There were no other significant main or interaction effects for System.

## 4. Discussion

The aim of this study was to evaluate the test-retest reliability, concurrent validity, and face validity of 10MWT gait parameters derived from AR data in people with Parkinson’s disease against lab-based and clinical references. We expected and found that 10MWT parameters quantified from AR data were reliable for test-retest, agreed well with those derived from reference systems, and varied in a-priori known directions over speed conditions. We further found, as expected, that the between-systems agreement was better than the within-system agreement over repetitions for all systems alike. Also, within-system differences in 10MWT parameters over speed conditions were, as expected, much greater in magnitude than the between-systems biases observed for some gait parameters.

### 4.1. Test-Retest Reliability and Concurrent Validity: A Comparison of Within-System and Between-Systems Agreement Statistics

When comparing 10MWT completion times and gait parameters over test repetitions, we observed that ICC values were good to excellent, yet with systematic biases (primarily for the CWS condition) and with wide limits of agreement (cf. Table 2). The ICC-values for completion time of the Comfortable Walking Speed condition were the lowest of all, for all three systems alike (~0.8), likely due to the significant bias of ~0.4 s between repetitions (see next section). The between-systems agreement statistics (Table 3) were better than the within-system test-retest agreement statistics (for all three systems alike), as evidenced by narrower between-systems limits of agreement than within-system limits of agreement over repetitions as well as by ICC values closer to 1 for the between-systems case (compare Table 2 and Table 3). This observation may suggest that 10MWT completion time, step length, cadence, and maximal gait speed can be interchangeably derived from AR data and lab-based (here Interactive Walkway) and clinical (here stopwatch) reference systems. However, in doing so, we should be conscious of the relatively poor within-system test-retest repeatability statistics (Table 2) for all systems, which may potentially hinder the detection of differences in 10MWT completion time, step length, cadence, and maximal gait speed between groups or over conditions (see next section).

### 4.2. Effects of Speed on 10MWT Parameters: A Comparison of Face Validity and Bias Results

We varied walking speed by instructing participants to complete the 10MWT at self-selected comfortable and fast-but-safe speeds. We found significant differences between these two conditions for all systems and for all parameters in a-priori known directions [9,10]. That is, test completion time decreased (~1.6 s faster) while step length (+8 cm), cadence (+14 steps/min), and maximal gait speed (+35 cm/s) all increased from comfortable to fast-but-safe speed conditions (Table 3, Figure 4 and Figure 5). These differences in a-priori known directions were much greater in magnitude than between-systems biases observed for some of the parameters (Table 3).

Also, the within-system biases observed over repetitions for the CWS condition are interesting in that regard (Table 2): we found a significant decrease in 10MWT completion time of ~0.4 s over trial repetitions. Interestingly, this systematic difference in completion time (roughly 25% of the difference observed over speed conditions) may be viewed as another test-case for face validity: this faster trial completion indeed resulted in significant biases in gait parameters in a-priori known directions, that is, ~2 cm longer step length (for both AR and IWW; Table 2), ~1.7 steps/min increase in cadence (for AR only; Table 2) and an increase of ~5 cm/s in maximal gait speed (for AR only; Table 2). This unintended, more subtle face validity evaluation suggests that not only large but also smaller differences in gait parameters derived from AR data may be detected with reasonable sensitivity. Thus, the relatively larger variability seen over repetitions (than between systems) did not negate the discriminative ability of step length, cadence, and gait speed derived from AR data for between-condition differences (between speeds), which further demonstrates face validity.

### 4.3. Prospects of Deriving Gait Parameters from AR Data

The use of AR glasses to support supervised clinical tests has gained traction, with studies showing the validity of data from AR devices like HoloLens 1 and HoloLens 2 for mobility assessments. For example, Sun et al. (2019) demonstrated that the HoloLens 1 could guide users through functional mobility tests, such as the Timed Up and Go (TUG) test, with performance metrics comparable to those seen with inertial measurement units (IMUs) [27]. Similarly, Koop et al. (2020) and Geerse et al. (2020) validated HoloLens 1 data for spatiotemporal gait parameterization, showing its reliability for measuring step length, cadence, and gait speed in both healthy individuals and people with Parkinson’s disease. These studies highlighted excellent test-retest reliability and between-systems agreement for these parameters [8,28]. More recently, Koop et al. (2022) validated HoloLens 2 data against 3D motion-capture systems, demonstrating its capacity to accurately measure gait parameters during walking and TUG tasks, with errors below 5% for key metrics like step length, stride length, and turning velocity [29]; see also van Bergem et al. (2024) for a recent reliability and validity evaluation of HoloLens 2 and Magic Leap 2 AR data for TUG parameterization [30]. All these studies, including this one, provide converging evidence that gait parameters can be derived reliably and validly from AR data, especially when registered with state-of-the-art AR glasses.

This study extends our prior work [8] by addressing critical gaps. First, we implemented a novel, standalone AR realignment method that is no longer reliant on an external reference system (e.g., Interactive Walkway), therefore enhancing the utility of AR data collection for independent gait assessments. Second, this study expands the 8-m walkway to a widely recognized clinical standard 10-m walk test, increasing its clinical relevance. Third, we assessed the face validity of AR-derived gait parameters in people with Parkinson’s disease, broadening the applicability and demonstrating its value in monitoring the disease’s progression. Moreover, this is the first study to introduce AR-based differentiation of left and right steps, potentially enabling a more detailed assessment of asymmetries in gait, particularly applicable in populations with neurological conditions.

AR glasses represent yet another new wearable device to parameterize gait alongside already established wearable technologies, such as inertial and pressure sensors [31,32]. While these devices have already shown their reliability and validity for measuring aspects of gait [33,34], they share a major limitation. They cannot enforce standardized test conditions, making them less suitable for automated unsupervised assessments. In contrast, AR glasses provide a unique combination of portability, inside-out tracking, standalone functionality, and the potential of real-time AR guidance. This may help ensure consistent adherence to assessment conditions, even in unsupervised environments, making AR glasses especially useful for remote, automated applications. Beyond traditional gait assessments, AR glasses may also allow clinicians to monitor the mobility of a patient at home over time during gamified AR exercises, offering essential insights for clinicians to manage the disease [6]. These and other digital health technology prospects of AR glasses, which go beyond just being another sensor for collecting data during a 10MWT, will be discussed in more detail next.

### 4.4. Prospects of AR Glasses Beyond Data Collection

The prospect of AR glasses as a digital health technology is broader than just for data collection. By using AR glasses, patients could receive AR guidance to potentially perform self-administered mobility assessments at home or in other settings, which could significantly reduce the burden on healthcare professionals [35]. That is, the use of holographic content allows for real-time guidance, ensuring that test constraints (such as intended walking speed and distance) are met and that test execution remains standardized (e.g., instructions, start indication), even without the direct supervision of a clinician. This self-administered digital health technology model could be especially beneficial for persons with Parkinson’s disease, a condition notorious for its fluctuations in motor symptoms over time: being able to more frequently assess gait makes the assessments less susceptible to fluctuations (i.e., they are averaged out over multiple assessments) compared to the occasional 10MWT snapshots taken during a clinical visit.

A third prospect beyond facilitating clinical assessments is that AR glasses may derive continuous mobility data that may allow for tracking changes in mobility over time. There may be different valuable use cases for this. First, as AR digital health technologies start to find their way into (remote) rehabilitation [36,37,38,39], such monitoring could become an integral part of AR rehabilitation interventions. As an example, we are currently studying the (clinical) feasibility and effectiveness of remotely prescribed AR exercises for improving gait and balance in people with Parkinson’s disease [40], training 6 weeks independently at home, using a variety of different gamified AR exercises. Some of these AR exercises comprise a strong gait component, such as Puzzle Walk (puzzle pieces are scattered in the living room and need to be collected and placed on an easel) and Smash (people need to walk back and forth between two plinths to punch AR objects to pieces). Previous research has shown that gamification can improve adherence to rehabilitation programs and enhance outcomes in mobility and balance interventions [41]. Next to adherence and game-play performance scores, we also record 3D position and orientation data from the AR glasses, from which we intend to derive walking segments and associated gait parameters to be able to analyze functional progress during home-based rehabilitation.

A second use case may be AR cueing. Specifically, AR-derived gait parameters could support personalized AR cueing, for example by tailoring AR cue activation to a particular motor state (e.g., standing, walking, freezing) and AR cue characteristics (like intercue distances) to an individual’s step length, ideally in a speed-dependent manner given the known effects of speed on gait parameters [9,10]. The current face validity evaluation, including the unintended one described above, showed that gait parameterization from AR data is sensitive enough to detect alterations in gait parameters with walking speed. A requirement for adjusting AR cues to different walking speeds is that AR data must be processed in semi-real-time and that it must work in less-constrained situations than the 10MWT studied here. This is certainly a challenge, especially considering the effects of disease-specific symptoms like dyskinesia on AR positional data during walking and assumptions made regarding features in AR positional data representing the feet in space. Nevertheless, it is a challenge worth pursuing as the prospect of personalized AR cueing is that it may help improve and modify gait [42,43,44] and reduce or prevent freezing episodes [42,45,46]. Existing studies have already demonstrated the potential effectiveness of AR cueing in enhancing gait patterns in people with Parkinson’s disease, despite AR technology still being in development at that time [47,48]. The integration of left-right step classification, demonstrated to have excellent concurrent validity in this study, could enhance the AR cueing experience for persons with Parkinson’s disease as it would allow for AR cue presentation in spatiotemporal alignment with their ongoing gait pattern.

### 4.5. Limitations and Future Work

This study has some limitations that should be acknowledged. First, head movements caused by dyskinesia in people with Parkinson’s disease significantly affect the accuracy of AR gait parameterization, as it heavily relies on head displacements. Future research could investigate approaches to mitigate noise in the head positional data due to dyskinetic movements, for example, by applying more advanced filtering techniques. Second, while this study introduces the differentiation of left and right steps, the investigation of stride time variability, a gait measure significantly associated with fall frequency, UPDRS-scores, ADL abilities, motor function, and responsiveness to levodopa [49], was not feasible due to limitations in the reference system. The Interactive Walkway, which was used as the reference system in this study, excels in spatial measurements but has limitations in temporal measurements, such as stride time and cadence. Future studies could aim to evaluate AR-derived temporal gait parameters against a gold-standard system, such as high-fidelity motion capture, to confirm their validity and fully explore their potential for clinical applications.

## 5. Conclusions

This study demonstrates that AR glasses, specifically the HoloLens 2 and Magic Leap 2, can reliably and validly derive various gait parameters (e.g., step length, cadence, gait speed, and left-right steps) during the 10MWT in individuals with Parkinson’s disease. Compared to previous studies, we introduced methodological advancements, including a standalone realignment method for AR data, the use of a widely recognized clinical test (10MWT), and the validation of novel left-right step differentiation. These findings underscore the potential of data collected from AR glasses for mobility assessments, offering promising applications in remote rehabilitation, continuous mobility monitoring, and personalized cueing.

## Figures and Tables

**Table 1:** Participants characteristics.

Characteristics	Mean ± SD [Range] or No.
Age (years)	66.9 ± 8.9 [51–82]
Weight (kg)	79.1 ± 9.9 [59.0–92.9]
Height (cm)	176.1 ± 10.3 [154–191]
Sex, male/female	15/5
Time since diagnosis (years)	8.0 ± 4.8 [1–20]
Modified Hoehn and Yahr [11] stage, 2/2.5	12/8
Freezing of gait, yes/no *	10/10
MDS-UPDRS III score ^1^	31.7 ± 11.9 [13–61]
Fall history (number of falls over the previous year)	2.8 ± 3.5 [0–10]

^1^ MDS-UPDRS III: MDS-unified Parkinson’s disease rating scale part III [12]. * The presence of freezing of gait is defined by a non-zero score on the New Freezing of Gait Questionnaire [13].

**Table 2:** Test-retest reliability: Absolute-agreement statistics bias, Limits of Agreement (LoA) and ICC for test completion time, mean step length, cadence, and maximum gait speed for both CWS and FWS conditions, separately for Augmented Reality (AR), Interactive Walkway (IWW), and Stopwatch (SW) systems.

Condition	Parameter	System	Trial 1 Mean ± SD	Trial 2 Mean ± SD	Bias (95% LoA)	t-Statistics	ICC(A,1)
**CWS**	**Completion time (s)**	**AR**	8.56 ± 1.10	8.19 ± 0.87	−0.36 (−1.38 0.65)	*t*(19) = 3.14, *p* = **0.005**	0.813
		**IWW**	8.66 ± 1.29	8.26 ± 0.89	−0.41 (−1.78 0.96)	*t*(19) = 2.60, *p* = **0.018**	0.757
		**SW**	8.98 ± 1.27	8.61 ± 1.11	−0.37 (−1.72 0.98)	*t*(19) = 2.38, *p* = **0.028**	0.801
	**Step length (m)**	**AR**	0.67 ± 0.10	0.69 ± 0.09	0.02 (−0.03 0.07)	*t*(19) = −3.05, *p* = **0.007**	0.948
		**IWW**	0.68 ± 0.10	0.70 ± 0.10	0.02 (−0.03 0.07)	*t*(19) = −3.48, *p* = **0.002**	0.950
	**Cadence (steps/min.)**	**AR**	109.6 ± 6.9	111.3 ± 7.3	1.69 (−3.80 7.18)	*t*(19) = −2.70, *p* = **0.014**	0.899
		**IWW**	110.8 ± 8.1	111.5 ± 6.9	0.71 (−5.32 6.73)	*t*(19) = −1.03, *p* = 0.316	0.917
	**Max. gait speed (m/s)**	**AR**	1.33 ± 0.16	1.38 ± 0.16	0.05 (−0.10 0.20)	*t*(19) = −2.78, *p* = **0.012**	0.848
		**IWW**	1.34 ± 0.16	1.38 ± 0.15	0.04 (−0.12 0.19)	*t*(19) = −2.05, *p* = 0.054	0.857
**FWS**	**Completion time (s)**	**AR**	6.93 ± 1.08	6.70 ± 0.99	−0.22 (−0.97 0.52)	*t*(19) = 2.64, *p* = **0.016**	0.915
		**IWW**	6.89 ± 1.12	6.77 ± 1.24	−0.11 (−1.17 0.94)	*t*(19) = 0.96, *p* = 0.350	0.897
		**SW**	7.15 ± 1.21	6.99 ± 1.11	−0.16 (−1.06 0.73)	*t*(19) = 1.60, *p* = 0.127	0.917
	**Step length (m)**	**AR**	0.75 ± 0.11	0.77 ± 0.10	0.02 (−0.04 0.08)	*t*(19) = −2.63, *p* = **0.017**	0.953
		**IWW**	0.76 ± 0.12	0.77 ± 0.11	0.01 (−0.05 0.08)	*t*(19) = −1.95, *p* = 0.066	0.952
	**Cadence (steps/min.)**	**AR**	123.8 ± 12.0	125.0 ± 12.5	1.18 (−8.16 10.53)	*t*(19) = −1.11, *p* = 0.281	0.923
		**IWW**	125.3 ± 12.7	126.0 ± 12.9	0.64 (−7.90 9.17)	*t*(19) = −0.65, *p* = 0.522	0.944
	**Max. gait speed (m/s)**	**AR**	1.69 ± 0.31	1.73 ± 0.26	0.04 (−0.23 0.30)	*t*(19) = −1.25, *p* = 0.228	0.887
		**IWW**	1.69 ± 0.29	1.74 ± 0.28	0.04 (−0.19 0.27)	*t*(19) = −1.63, *p* = 0.119	0.911

Significant biases are presented with the *p*-values in **bold**.

**Table 3:** Concurrent validity: Absolute-agreement statistics bias, Limits of Agreement (LoA), and ICC for test completion time (Augmented Reality [AR] vs. Stopwatch [SW] and AR vs. Interactive Walkway [IWW]) and the gait parameters mean step length, cadence, and maximum gait speed (AR vs. IWW), all separately for CWS and FWS conditions.

Condition	Parameter	Mean ± SD	Mean ± SD	Bias (95% LoA)	*t*-Statistics	ICC_(A,1)_
		**AR**	**SW**			
**CWS**	**Completion time (s)**	8.56 ± 1.10	8.98 ± 1.27	−0.42 (−0.46 1.29)	*t*(19) = −4.18, *p* = **0.001**	0.879
**FWS**	**Completion time (s)**	6.93 ± 1.08	7.15 ± 1.21	0.22 (−0.39 0.84)	*t*(19) = −3.18, *p* = **0.005**	0.946
		**AR**	**IWW**			
**CWS**	**Completion time (s)**	8.56 ± 1.10	8.66 ± 1.29	0.10 (−0.83 1.03)	*t*(19) = −0.97, *p* = 0.345	0.922
	**Step length (m)**	0.67 ± 0.10	0.68 ± 0.10	0.00 (−0.02 0.02)	*t*(19) = −0.73, *p* = 0.473	0.994
	**Cadence (steps/min.)**	109.6 ± 6.8	110.8 ± 8.1	1.24 (−3.31 5.80)	*t*(19) = −2.39, *p* = **0.027**	0.942
	**Max. gait speed (m/s)**	1.33 ± 0.16	1.34 ± 0.16	0.01 (−0.04 0.06)	*t*(19) = −1.94, *p* = 0.067	0.985
**FWS**	**Completion time (s)**	6.93 ± 1.08	6.89 ± 1.25	−0.04 (−0.36 0.28)	*t*(19) = 1.03, *p* = 0.318	0.989
	**Step length (m)**	0.75 ± 0.11	0.76 ± 0.12	0.00 (−0.02 0.03)	*t*(19) = −1.36, *p* = 0.190	0.993
	**Cadence (steps/min.)**	123.8 ± 12.0	125.3 ± 12.7	1.51 (−1.49 4.50)	*t*(19) = −4.40, *p* < **0.001**	0.985
	**Max. gait speed (m/s)**	1.69 ± 0.31	1.69 ± 0.29	0.00 (−0.10 0.10)	*t*(19) = 0.01, *p* = 0.990	0.985

Significant biases are presented with the *p*-values in **bold**.

## Data Availability

All data is made available as Data files in the Supplementary Material.

## Supplementary Materials

The following supporting information can be downloaded at https://www.mdpi.com/article/10.3390/s25041230/s1: Datafile S1: AR data and outcomes; Datafile S2: Interactive Walkway data and outcomes; Datafile S3: Stopwatch outcomes. All data files are mat files.
